# Cytidine analogs are synthetic lethal with base excision repair default due to MBD4 deficiency

**DOI:** 10.1038/s41698-022-00326-z

**Published:** 2022-11-02

**Authors:** Thomas Chabot, Fariba Nemati, Aurélie Herbette, Alexandre Demeyer, Stéphane Dayot, Olivier Ganier, Samar Alsafadi, Sophie Gardrat, Pascale Mariani, Marie Luporsi, Maxime Corbé, Vincent Servois, Nathalie Cassoux, Didier Decaudin, Sergio Roman Roman, Elaine Del Nery, Sophie Piperno-Neumann, Marc-Henri Stern, Manuel Rodrigues

**Affiliations:** 1 Inserm U830, DNA Repair and Uveal Melanoma (D.R.U.M.), Equipe labellisée par la Ligue Nationale Contre le Cancer, Institut Curie, PSL Research University, Paris, 75005 France; 2 Laboratory of Preclinical Investigation, Translational Research Department, Institut Curie, PSL Research University, Paris, 75005 France; 3 Biophenics, PICT-IBiSa, Translational Research Department, Institut Curie, PSL Research University, Paris, France; 4 Uveal Melanoma Group, Translational Research Department, Institut Curie, PSL Research University, Paris, 75005 France; 5 Department of Biopathology, Institut Curie, PSL Research University, Paris, 75005 France; 6 Department of surgical oncology, Institut Curie, PSL Research University, Paris, 75005 France; 7 Department of Nuclear Medicine, Institut Curie, PSL Research University, Paris, 75005 France; 8 LITO Laboratory INSERM U1288, Institut Curie, Orsay, 91440 France; 9 Department of Medical Imaging, Institut Curie, PSL Research University, Paris, 75005 France; 10 Department of Ocular Oncology, Institut Curie, Paris, 75005 France; 11 Faculty of Medicine, University of Paris Descartes, Paris, 75005 France; 12 Department of Medical Oncology, Institut Curie, PSL Research University, Paris, 75005 France

**Keywords:** Chemotherapy, Phenotypic screening, Genomic instability

## Abstract

Inactivating mutations of *MBD4* have been reported in subsets of various tumors. A deficiency of this DNA glycosylase, recognizing specifically T:G mismatch resulting from the deamination of methyl-cytosine, results in a hypermutated phenotype due to the accumulation of CpG>TpG transitions. Here, we hypothesize that the difference in DNA metabolism consecutive to *MBD4* deficiency may result in specific cytotoxicities in MBD4-deficient tumor cells in a synthetic lethality fashion. After a large-scale drug repurposing screen, we show in two isogenic *MBD4* knock-out cell models that the inactivation of *MBD4* sensitizes cancer cells to cytidine analogs. We further confirm the exquisite activity of gemcitabine in an *MBD4*-deficient co-clinical model as (i) it completely prevented the development of an *MBD4*-deficient uveal melanoma patient-derived xenograft and (ii) treatment in the corresponding patient resulted in an exceptional tumor response. These data suggest that patients harboring MBD4-deficient tumors may be treated efficiently by cytidine analogs.

## Introduction

The genome is constantly under assault of intrinsic and extrinsic genotoxic agents, which may lead to ageing, neurodegenerative disorders, or cancers. According to the nature of DNA damage, different DNA repair mechanisms participate in maintaining genome integrity. Among them, the base excision repair (BER) pathway takes in charge lesions that typically result from deamination, oxidation, or alkylation of bases^1^. BER is initiated by DNA glycosylases (e.g., MBD4, TDG, UNG, OGG1) that recognize and excise the damaged base (e.g., T:G, U:G, 8oxo-G:C mismatches), leaving an abasic site that is further processed either by short or long-patch repair pathways^1^. *MBD4* encodes the only human protein having a monofunctional DNA glycosylase domain (GD) associated with a methyl-CpG binding domain (MBD)^2^. The GD of MBD4 cleaves the *N*-glycosidic bond of thymidine in T:G mismatches that result from the spontaneous deamination of 5-methylcytosines (5mC) in a 5mCpG dinucleotide context^3^. This site is then cleaved by the apurinic/apyrimidinic endonuclease 1 (APE1), initiating the BER pathway. The missing base position is filled by DNA polymerase β/δ action and the strand continuity is restored by DNA ligase LIG1/3^4^. Consequently, *Mbd4*-null mice present a 2–3-times higher rate of somatic CpG>TpG transitions^5^. Similarly, a dramatic increase in the rate of CpG>TpG mutations is observed when *MBD4* is inactivated in cancer cells^6–9^. Although MBD4 presents a specific enzymatic activity recognizing such mismatches, thymine DNA glycosylase (TDG) can also be implicated in this process^10^.

*MBD4* inactivation has been identified in different cancer types, such as acute myeloid leukemia, colorectal carcinoma, glioma, spiradenocarcinoma, and uveal melanoma (UM)^7,8,11,12^. UM is the most common primary intraocular tumor^13^. When metastatic, treatment options, including cytotoxic chemotherapies, immune checkpoint inhibitors, and targeted agents, are associated with extremely low response rates. Recently, tebentafusp, a novel bispecific T-cell engager, demonstrated a clinical benefit but was limited to HLA A02:01-positive patients (45% of Caucasian patients)^14^. Although we expect that patients harboring hypermutated *MBD4*-deficient metastatic tumors will benefit from immune checkpoint inhibition (ICI), secondary resistance to ICI is anticipated, as already reported^7^. These data highlight the need to establish new treatments.

The biological consequences of MBD4 inactivation impact BER and, subsequently multiple other DNA metabolism processes from replication to methylation. Therefore, we hypothesized that MBD4-deficient tumors could be more sensitive to pharmacological agents targeting the impacted biological processes. Therefore, we performed a large-scale drug repurposing screen for drugs that could be synthetic and lethal with *MBD4* deficiency. Among candidate drugs, two cytidine analogs that are commonly used in oncology, gemcitabine or cytarabine, selectively target *MBD4*-deficient cells in vitro in two isogenic MBD4 cell lines, in vivo in a xenograft derived from an *MBD4*-deficient UM patient (PDX) and ultimately in an *MBD4*-deficient metastatic UM patient although gemcitabine is usually inefficient in UM.

## Results

### A drug repurposing screen reveals cytidine analogs as candidates to target *MBD4*-deficient models

**Fig. 1:**
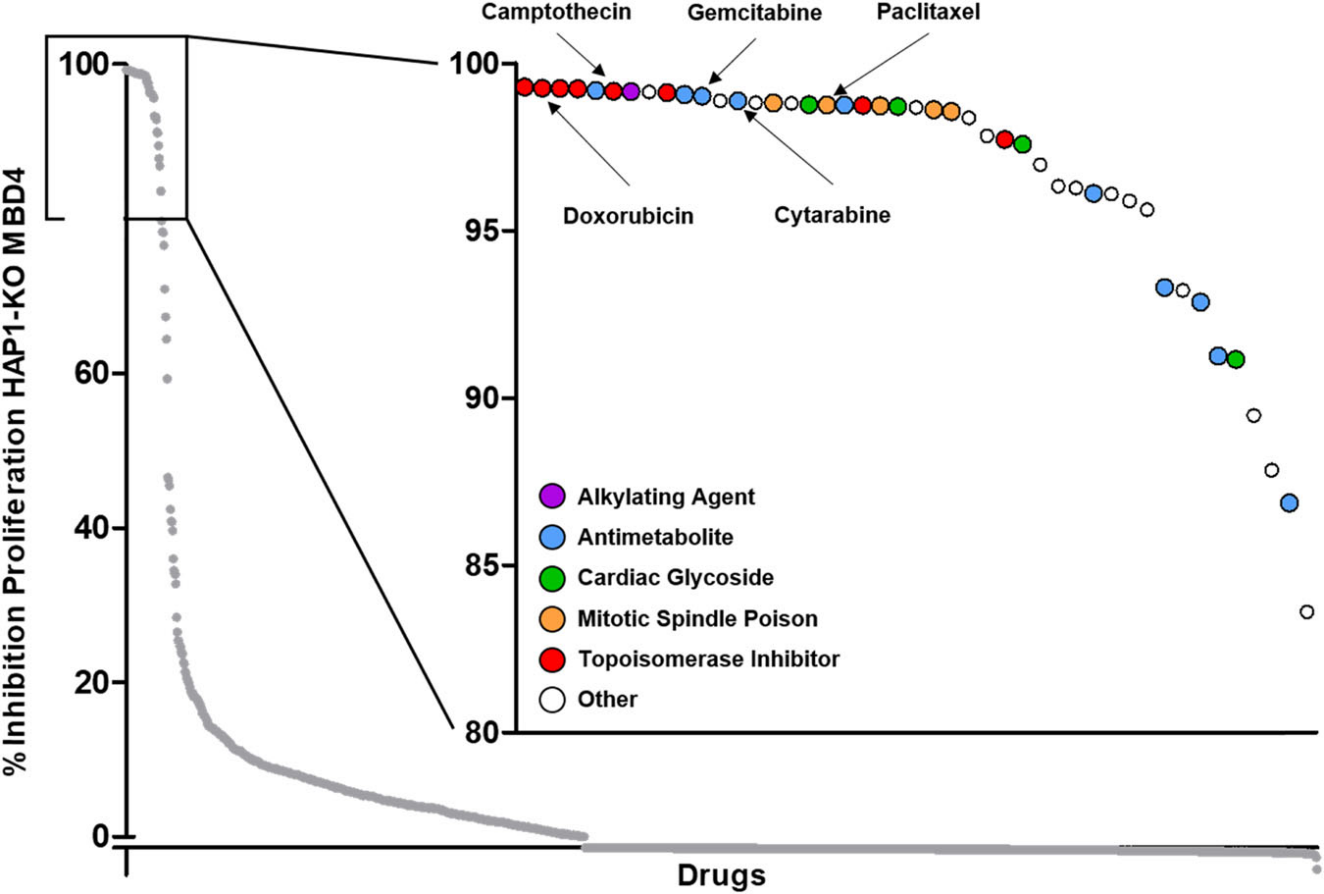
Screening of the Prestwick Chemical Library corresponding to a collection of 1520 drugs on cell proliferation in the HAP1-KO MBD4 cell line. Scatter plot of the percentage of inhibition of proliferation of HAP1-KO MBD4 cell line treated with a focus on compounds that reduce the proliferation of HAP1-KO MBD4 at over 80%. DMSO was used for the negative control.

To search for drugs targeting MBD4-deficient tumors, we proceeded to a large-scale screen of 1520 compounds. Our screening strategy aimed at identifying the effect of these agents in inhibiting the proliferation of the commercially available isogenic MBD4-deficient (HAP1-KO MBD4) cell line at 1 µM. Forty-five of these compounds inhibited HAP1-KO MBD4 proliferation by 80% or more (Fig. 1 and Supplementary Table 1). Among the most active of these molecules, we observed the presence of chemotherapeutic molecules, such as topoisomerase inhibitors (camptothecin, a topoisomerase I inhibitor, and doxorubicin, a topoisomerase II inhibitor), antimetabolite drugs (cytidine analogs gemcitabine and cytarabine), and mitotic spindle poisons such as paclitaxel. We then compared the IC_50_ cell viability of HAP1-KO MBD4 in comparison with isogenic wild-type cells to search for differential sensitivity to commonly used agents from these families.

**Fig. 2:**
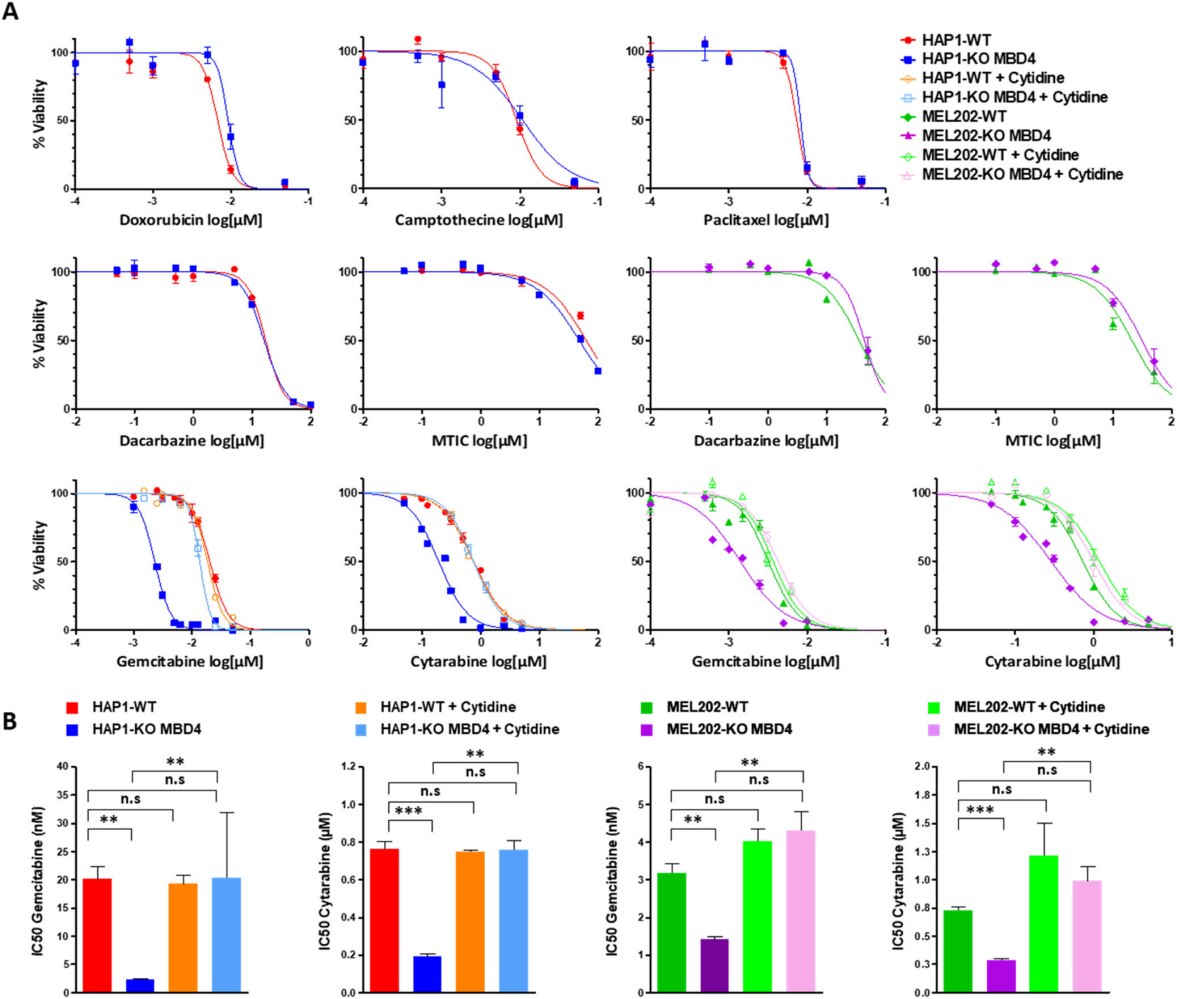
MBD4-deficient cell lines are more sensitive to gemcitabine and cytarabine than MBD4-proficient counterparts. **A** Cell count assay assessing cell viability of HAP1 and MEL02 isogenic cell lines. **B** IC_50_ for each culture condition of HAP1 and MEL202 isogenic cell lines was obtained from a dose-response curve (*n* = 3, mean ± SD). Cell count and IC_50_ were calculated from three independent assays for each condition. Cytidine addition (2 µM final) or not is indicated. n.s non-significant; **P < 0.01; ***P < 0.001; unpaired two-sided Student’s *t*-test).

We treated isogenic HAP1-KO MBD4 and HAP1-WT cell lines with camptothecin, doxorubicin, gemcitabine, cytarabine, paclitaxel, and dacarbazine, an alkylating agent commonly used to treat uveal melanoma patients (together with its active form, MTIC). In control culture conditions, HAP1-KO MBD4 and HAP1-WT cell lines presented similar proliferation rates (Supplementary Fig. 2). We did not detect any significant difference in IC_50_ between HAP1-KO MBD4 and HAP1-WT upon camptothecin, doxorubicin, paclitaxel, dacarbazine or MTIC treatment (Fig. 2A). By contrast, we observed a ten-fold increase of sensitization to gemcitabine in HAP1-KO MBD4 as compared with HAP1-WT (IC_50_: 2.3 nM ± 0.1 versus 20.1 nM ± 1.5, respectively; *P* = 2.82 × 10^−3^) (Fig. 2A, B), which was further confirmed by MTT cell viability and proliferation assay (IC_50_: 3.7 nM ± 0.9 versus IC_50:_ 15.3 nM ± 2.7, respectively; *P* = 2.31 × 10^−4^). We noticed a similar increase of sensitization to cytarabine of HAP1-KO MBD4 compared to WT (IC_50_: 0.19 µM ± 0.02 versus 0.76 µM ± 0.06, respectively; *P* = 6.45 × 10^−4^) (Fig. 2A, B), confirmed by MTT assay (IC_50_: 0.55 µM ± 0.08 versus IC_50_: 0.92 µM ± 0.07; *P* = 9.5 × 10^−3^). Importantly, the addition of cytidine to the medium prevents the sensitization of HAP1-KO MBD4 to gemcitabine treatment (IC_50_: 2.3 nM ± 0.1 versus 13.5 nM ± 0.9, without and with cytidine supplementation, respectively; *P* = 4.21 × 10^−3^), whereas IC_50_ of parental HAP1-WT remained unchanged (IC_50_: 20.1 nM ± 1.5 versus 18.4 ± 1.5, without and with cytidine supplementation, respectively; *P* = 0.34) (Fig. 2A, B). Similarly, cytidine supplementation rescued HAP1 death by preventing apoptosis (from 30.5% of annexin V positive in HAP1-KO MBD4 without cytidine to 20.8% with cytidine, respectively; *P* = 0.004; Supplementary Fig. 3). We also observed that the addition of cytidine to HAP1-KO MBD4 cells resulted in a similar IC_50_ than in HAP1-WT cells (*P* = 0.44). Cytidine supplementation resulted in the same reversal effect for cytarabine treatment in HAP1-KO MBD4 (IC_50_: 0.19 µM ± 0.02 versus 0.75 µM ± 0.07, without and with cytidine supplementation, respectively; *P* = 1.81 × 10^−3^), whereas parental HAP1-WT sensitivity remained unchanged (IC_50_: 0.76 µM ± 0.06 versus 0.74 µM ± 0.07, without and with cytidine supplementation, respectively; *P* = 0.33) (Fig. 2A, B). Again, following the addition of cytidine to HAP1-KO MBD4 media, the IC_50_ was similar to that of HAP1-WT upon cytarabine treatment, combined or not with cytidine (*P* = 0.32).

To test the consequences of *MBD4* deficiency in a UM context, we genetically engineered by CRISPR an isogenic UM cell line deficient for MBD4 (MEL202-KO MBD4) or not (MEL202-WT) and assessed their sensitivity to cytidine analogs. We did not detect any difference between the two isogenic MEL202-derived cell lines upon dacarbazine treatment or its metabolite MTIC (Fig. 2A, B). As observed for HAP1, we observed a significantly higher sensitivity of MEL202-KO MBD4 to gemcitabine in comparison to MEL202-WT (IC_50_: 1.4 nM ± 0.1 versus IC_50_: 3.2 nM ± 0.3; *P* = 5.09 × 10^−3^) (Fig. 2A, B), and similarly for cytarabine (IC_50_: 0.29 µM ± 0.02 versus IC_50_:0.73 µM ± 0.03; *P* = 5.52 × 10^−4^) (Fig. 2A, B). We again noticed a prevention of sensitivity to gemcitabine treatment with cytidine supplementation (IC_50_: 1.4 nM ± 0.1 versus 4.3 nM ± 0.6, without and with cytidine supplementation, respectively; *P* = 3.14 × 10^−3^), whereas cytotoxicity on parental MEL202-WT remained unchanged (IC_50_: 3.2 nM ± 0.3 versus 3.7 ± 0.6, without and with cytidine supplementation, respectively; *P* = 0.13) (Fig. 2A, B). Treatment with cytarabine showed similar results on MEL202-KO MBD4 (IC_50_: 0.29 µM ± 0.02 versus 0.99 µM ± 0.2, without and with cytidine supplementation, respectively; *P* = 4.87 × 10^−3^), while parental MEL202-WT sensitivity remained unchanged (IC_50_: 0.73 µM ± 0.03 versus 1.19 ± 0.2, without and with cytidine supplementation, respectively; *P* = 0.43) (Fig. 2A, B).

In summary, *MBD4* deficiency increased the sensitivity of cancer cell lines to cytidine analogs (gemcitabine and cytarabine), but not to other classes of chemotherapeutic agents, including dacarbazine, commonly used in UM treatment. We thus selected cytidine analogs for further in vitro experiments.

### Cytidine analogs and cell death in an *MBD4*-deficient context

**Fig. 3:**
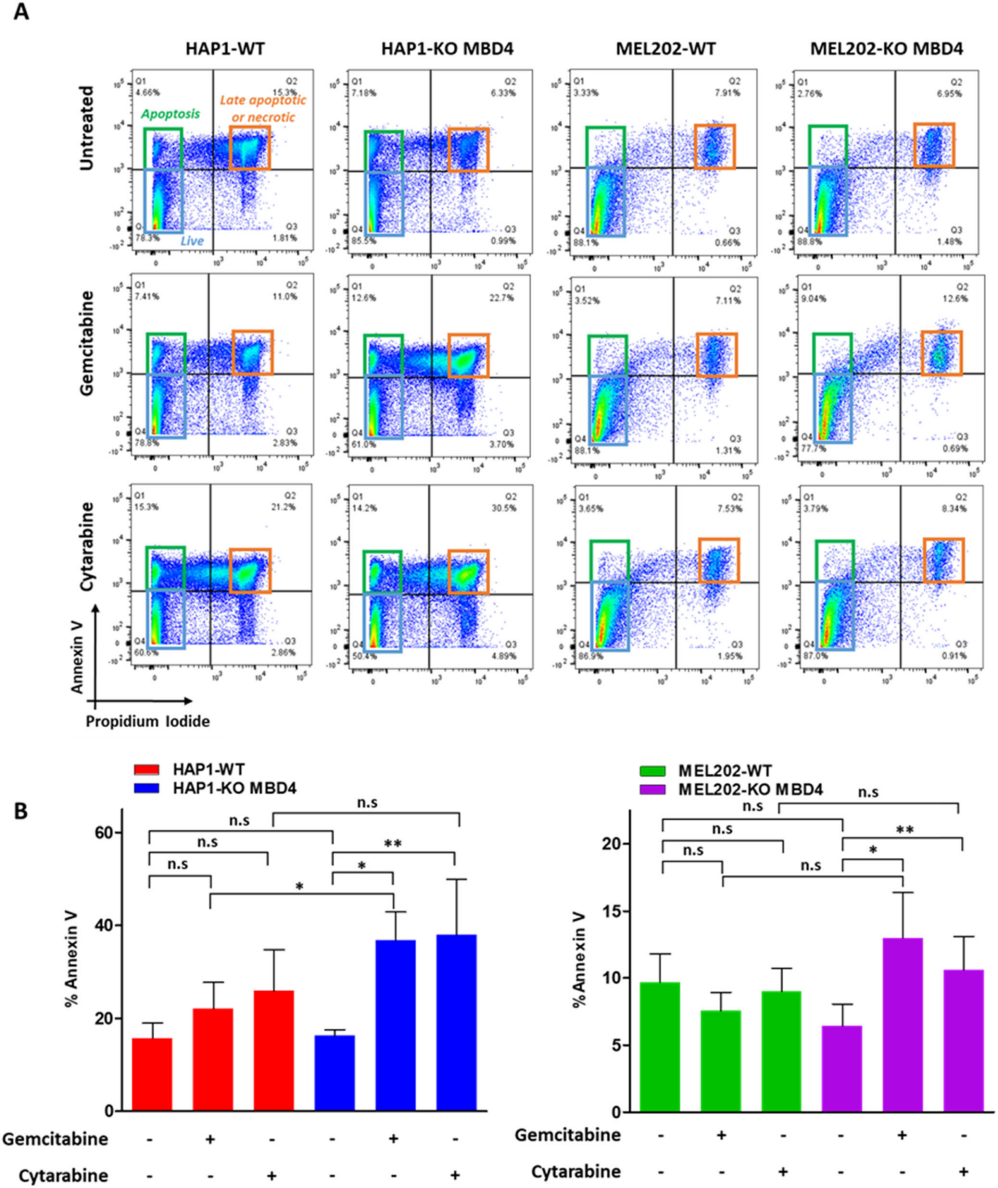
Gemcitabine and cytarabine increase cell death of MBD4-deficient cell lines as compared with proficient cell lines. **A** Analysis of the scatter plot. Annexin V/Propidium Iodide double-negative cells correspond to live cells (blue square), Annexin V positive/Propidium Iodide negative cells correspond to apoptotic cells (green square), and Annexin V/Propidium Iodide double-positive cells correspond to late apoptotic and necrotic cells or undetermined population (orange square). Scatter plots represent a representative experiment among three carried out independently. **B** Percentage of HAP1 and MEL202, MBD4-proficient (red and green columns) and deficient (blue and violet columns) Annexin V positive cells from three independent assays (mean ± SD; n.s non-significant; *P < 0.05; **P < 0.01; unpaired two-sided Student’s *t*-test).

We next evaluated the impact of cytidine analogs on cell death. Both untreated HAP1 isogenic cell lines (HAP1-WT and HAP-KO MBD4) showed similar proportions of annexin V positive (whether positive or not for propidium iodide) cells (Fig. 3A), whatever their *MBD4* status (15.8% ± 3.3 versus 16.2% ± 1.3 in HAP1-KO MBD4 and parental HAP1-WT, respectively; *P* = 0.43) (Fig. 3B). We observed a significant increase of cell death in HAP1-KO MBD4 following treatment with gemcitabine (from 16.2% ± 1.3 to 36.8% ± 6.2; *P* = 1.6 × 10^−2^) but not in parental HAP1-WT cells (from 15.8% ± 3.3 to 22.1% ± 5.6; *P* = 0.15) (Fig. 3A, B). Similarly, treatment with cytarabine induced a trend towards more cell death in the MBD4-deficient context (Fig. 3A), albeit not significant (from 16.2% ± 1.3 to 34.7% ± 14.5; *P* = 8.3 × 10^−2^ *versus* from 15.8% ± 3.3 to 25.9% ± 8.8; *P* = 9.6 × 10^−2^, for HAP1-KO MBD4 and HAP1-WT respectively) (Fig. 3B).

Both MEL202-derived cell lines (MEL202-WT and MEL202-KO MBD4) showed similar baseline proportions of annexin V positive and/or propidium iodide positive cells, whatever their *MBD4* statuses (from 6.4% ± 3.6 *versus* 9.7% ± 4.2 in MEL202-KO MBD4 and MEL202, respectively; *P* = 0.15) (Fig. 3A, B). However, while we did not observe an annexin V positive/propidium iodide negative population in any MEL202 cell line (Fig. 3A), following treatment with gemcitabine we observed a significant increase of cell death in MEL202-KO MBD4 (from 6.4% ± 3.6 to 12.9% ± 5.5; *P* = 3.6 × 10^−2^) but not in parental MEL202-WT cells (from 9.7% ± 4.2 to 7.6% ± 3.1; *P* = 0.27) (Fig. 3B). Similarly, treatment with cytarabine resulted in a more important increase of cell death in the MBD4-deficient context albeit not significant (from 6.4% ± 3.6 to 10.6% ± 5.2; *P* = 8.0 × 10^−3^ versus from 9.7% ± 4.2 to 9.1% ± 3.8; *P* = 0.42, respectively for MEL202-KO MBD4 and MEL202-WT) (Fig. 3A, B). We conclude that cytidine analogs tend to increase cell death in MBD4-deficient cell lines as compared with wild-type cell lines.

### Response to gemcitabine in an MBD4-deficient UM PDX and corresponding patient

**Fig. 4:**
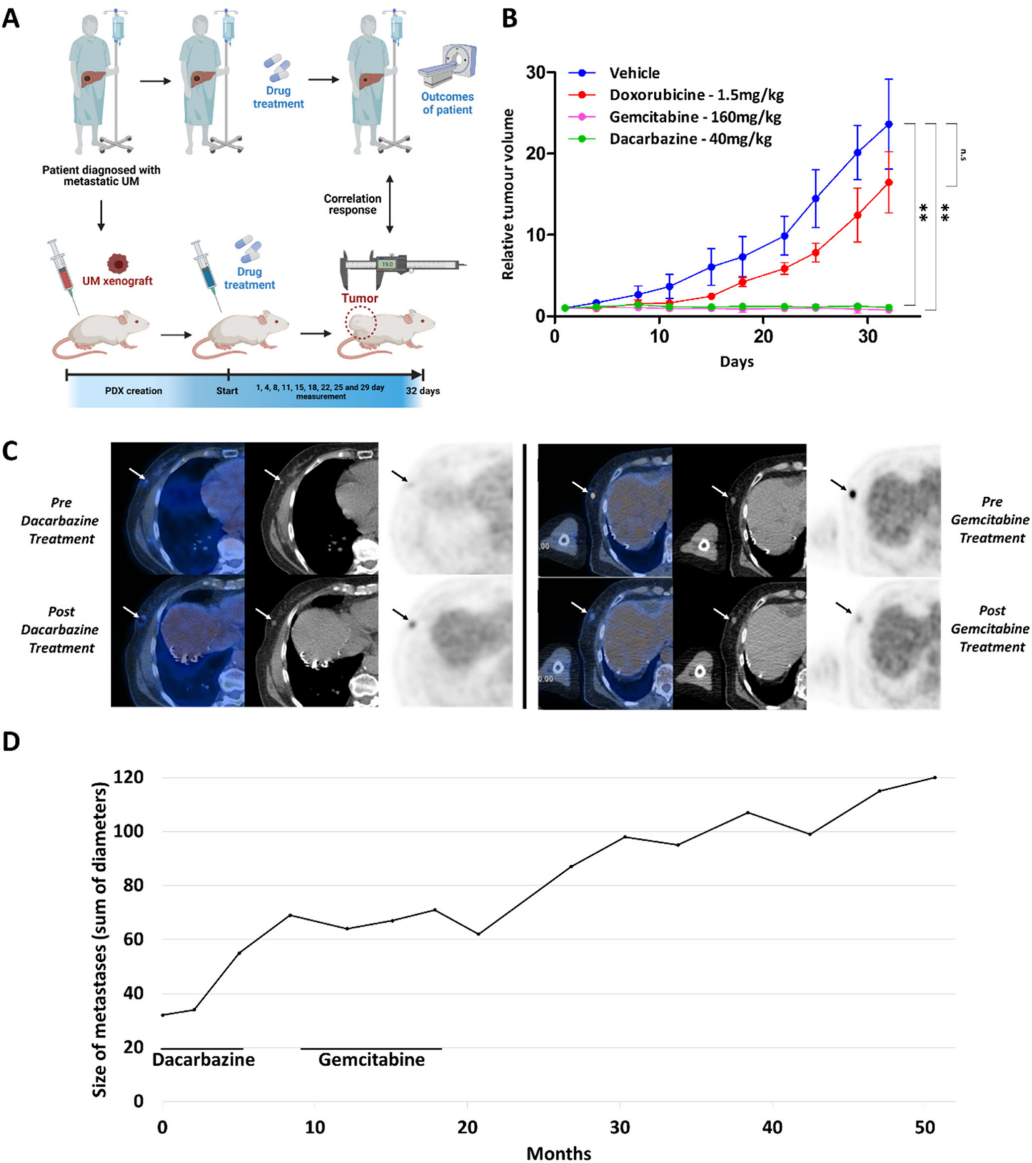
Activity of gemcitabine in an MBD4-deficient UM patient-derived xenograft (PDX) model and in the corresponding metastatic patient. **A** Schema of a co-clinical trial model between a PDX model and the patient from whom was derived the PDX. Created with BioRender.com. **B** Mice were treated with different drugs and tumor growth was assessed by measuring the relative tumor volume (RTV) for 32 days (*n* = 4 mice/drug, mean ± SD; n.s non-significant; **P < 0.01; unpaired two-sided Student’s *t*-test). **C** Positron emission tomography-computed tomography images before and after 5 months of dacarbazine (upper panel), and before and after 9 months of gemcitabine treatment (lower panel). Arrows show locations of metastatic lesions. **D** Evolution of the size of metastases during and after dacarbazine or gemcitabine treatment.

Having demonstrated in vitro the high sensitivity of MBD4 deficiency cancer cell lines to cytidine analogs, we then studied their effect in a co-clinical model. This model is based on the comparison of tumor progression between a PDX model and the patient from whom the PDX is derived (Fig. 4A). This co-clinical model was developed from a 77 years old patient who presented with a first hepatic recurrence from an *MBD4*-deficient UM, treated with margin-free liver surgery^7^. A patient-derived xenograft (PDX) was generated from this tumor sample.

In this first part of the co-clinical model, the PDX was treated with either gemcitabine, dacarbazine (standard-of-care chemotherapy for UM patients), or doxorubicin (standard-of-care chemotherapy in multiple other cancers but not in UM). Treatment either with dacarbazine or gemcitabine completely prevented the growth of the tumor in comparison to vehicle (−95.45% ± 1.5, *P* = 2.0 × 10^−3^ and –96.62% ± 1.1, *P* = 1.9 × 10^−3^ in 32 days, respectively), whereas doxorubicin did not (−30.29% ± 38.8, *P* = 0.18 and –36.68% ± 32.7, *P* = 0.16 in 32 days, respectively) (Fig. 4B).

In parallel, tumor progression was evaluated in the UM MBD4-deficient patient. The patient was successfully treated with pembrolizumab, an immune checkpoint inhibitor, for 22 months, as previously reported^7^. The patient subsequently presented progressive subcutaneous metastases leading to a standard second line with dacarbazine. Dacarbazine was inefficient as the sum of diameters of lesions increased by 26% in five months as assessed by computed tomography, with no major metabolic changes on 18F-FDG positron emission tomography (maximum standardized uptake values going from 4.1 and 1.4 for the two main lesions on baseline exam to 3.8 and 3.7 after dacarbazine, respectively). After a pause of three months, the patient then received gemcitabine, resulting in a metabolic response of metastatic lesions (Fig. 4C). Before gemcitabine, metastases were growing (the sum of their diameters increasing from 32 to 69 mm) then the growth of metastases stopped during gemcitabine treatment (with the sum of diameters oscillating between 62 and 71 mm; Fig. 4D). During the 9 months of treatment, maximum standardized uptake values of the main lesions reduced from 8.9 and 3.8 to 2.0 and 2.3. After gemcitabine, the size of metastases grew again (from 62 to 87 mm in 6 months to 120 mm at the last assessment). We show in this co-clinical model that the use of gemcitabine (a cytidine analog) leads to a dramatic response of *MBD4*-deficient UM on tumor progression.

## Discussion

We here show that targeting cytidine metabolism is a valid synthetic lethal strategy in an MBD4-deficient context. Importantly, we observed a higher sensitivity to cytosine analogs in an MBD4-deficient context not only in vitro in isogenic cell models, but also in vivo in an MBD4-deficient UM PDX and ultimately in the patient from whom the PDX was derived, a surprising response considering that UM is known to be highly chemo-resistant^15^. Gemcitabine has been used for the past 25 years for the treatment of multiple cancers, including breast, ovarian and pancreatic carcinomas. However, clinical trials in metastatic UM reported disappointing results in the early 2000s. The combination of gemcitabine with alkylating agent treosulfan in a phase II trial showed only one tumor response over 24 patients and seven stable diseases^16^. Similarly, no response and seven stable diseases were observed over 17 patients in another phase II trial combining gemcitabine, treosulfan, and cisplatin^17^. These poor responses with gemcitabine may be due to the rarity of MBD4-deficient UM patients in these cohorts, which needed to observe a real impact of gemcitabine. To our knowledge, the other cytidine analog we evaluated in vitro, cytarabine, has never been tested in UM patients and is rather prescribed for patients with hematological malignancies.

One hypothesis explaining the observed activity of gemcitabine and cytarabine is that these drugs act on a secondary step of BER, *i.e*. on the abasic site resulting from base excision. These analogs then compete with endogenous dCTP during DNA replication. Once integrated into the DNA in place of the deaminated methyl-cytosine, the cytidine analogs lead to the inhibition of the DNA polymerase, and to the termination of DNA synthesis^18,19^. In the absence of MBD4, BER of deaminated methylcytosines can probably still be processed through other non-specific glycosylases such as TDG, but less efficiently. Targeting an already hampered BER pathway would then result in an exacerbated decrease in the capacity to repair these lesions and the exquisite sensitivity of MBD4-deficient cells to these drugs. However, other processes may be implicated. It has been shown that in a mismatch repair-deficient setting, cells may be more sensitive to gemcitabine, although the mechanism is still unclear^20^. MBD4, then named MED1, was discovered as a member of a protein complex with MLH1, maintaining a possible link between BER and the mismatch repair machinery^2,21^. Following this hypothesis, MBD4 inactivation would not only slow down BER, but would also impede this link with the mismatch repair system, precluding a rescue repair mechanism. In contrast to gemcitabine, dacarbazine is a common option in the treatment of metastatic UM patients, albeit associated with a poor response rate^22,23^. Although the PDX model predicted a tumor response to dacarbazine, our experiments on isogenic cell lines did not show such sensitivity, and ultimately the patient did not respond to this drug. One plausible explanation is that dacarbazine randomly affects guanines all along the genome, contrary to gemcitabine, they directly target the first steps of BER by filling the abasic site, thus reducing the chances of observing a synthetic lethality phenomenon.

However, our study is limited to different levels. We might notice that HAP1 is a cell line derived from a patient with chronic myeloid leukemia, and that these cells might be inappropriate for a study about UM. The main advantage of this engineered HAP1 cell line was that this cell line is near-haploid, allowing an efficient, persistent inactivation of any gene with the CRISPR technology (MBD4 in the present case). Consequently, HAP1 have been widely used for multiple applications, including in >100 publications. Furthermore, the main objective of this work was precisely to identify therapies that are active in MBD4-inactivated tumors, independently from the cell context.

Another limitation of our work is the absence of a definitive explanation for the mechanism of action of cytidine analogs in MBD4-deficient cells. The mechanism of cell death may be different as early apoptotic cells could not be clearly identified in MEL202 recipients, whereas early apoptotic, annexin V-positive/PI-negative HAP1 cells could be observed.

Finally, the magnitude and duration of the effect of gemcitabine may seem modest in this patient when compared to other cancer therapies, probably because the disease was slowly evolving in this patient, but we must remind here that cytotoxic are usually inefficient in metastatic UM patients. Furthermore, gemcitabine was interrupted in this patient after 9 months of treatment not because of disease progression but because of a rare side effect suggesting that the effect may have lasted for a longer time if pursued. In fact, the previous line of treatment, dacarbazine, was associated with primary resistance and progressive disease. We believe this level of response is sufficient to consider treating with gemcitabine other patients with MBD4-deficient tumors.

Our study shows that targeting cytidine metabolism is a relevant strategy in MBD4-deficient tumors. Such tumors are probably rare, with mainly UMs, acute myeloid leukemias, colorectal carcinomas, and gliomas, but this list might be underestimated since the precise spectrum of MBD4-associated diseases remains to be defined. The scarce number of cases could limit the scope of our discovery, but such tissue-agnostic, biomarker-based therapeutic strategy has recently been validated with the approval of pembrolizumab in mismatch repair-deficient tumors^24^ and TRK inhibitors in *NTRK*-rearranged tumors, even though these fusions are present in less than 1% of common cancers^25^. We can also hypothesize that our results may have consequences beyond MBD4 deficiency, on the treatment of cancers that are proficient for MBD4. In these cases, we may expect that pharmacological inhibition of MBD4 together with cytidine analogs could lead to an increased sensitivity of MBD4-proficient tumors, opening new therapeutic avenues in MBD4-proficient tumors, whatever their histological types.

## Methods

### Cell lines

The HAP1-KO MBD4 cell line is commercially available (#CL-0108, Creative Biomart), modified from commercial HAP1-WT with a two base pair deletion in exon 2 of *MBD4* engineered by CRISPR-Cas9 (Supplementary Fig. 1A, C). MEL202-KO MBD4 was established from the primary UM cell line MEL202 (here named MEL202-WT)^26^. Briefly, MEL202-WT cells were modified by the CRISPR-Cas9 system with the integration of a puromycin box in exon 3 of *MBD4*. Integration sequence in MEL202-WT was confirmed by PCR with genomic DNA of MEL202-WT versus KO MBD4 by using PCR probe control (CATCATCAACACCCTCATCTTC and CAGATACCTATGGCAACATTTGG) and KO MBD4 specific (CAGATACCTATGGCAACATTTGG and CAGATACCTATGGCAACATTTGG) (Supplementary Fig. 1B, D). MEL202-KO MBD4 cell line and the PDX are available upon request to the authors. Cells were grown in Media 199 (M199; #11150059, Thermo Fischer Scientific)) supplemented with 10% Fetal Bovine Serum (FBS) at 37 °C and 5% CO_2_.

### Drug screening

The Prestwick Chemical Library (PCL) V3 was used for the drug screening. This library corresponds to a collection of 1520 small molecules, 98% of which being approved drugs (Food and Drug Administration, European Medicines Agency, and other agencies). All compounds were received in dimethyl sulfoxide (DMSO) as 10 mM stock solutions and represented a total of six 384-well plates. Cells were seeded on 384-well plates at 600 cells/well using a Multidrop Combi liquid dispenser (Thermo Fisher Scientific). We tested different DMSO concentrations for the selected cell density and confirmed minimal cell death with up to 0.05% DMSO. Twenty-four hours following cell seeding, compounds were first prediluted in M199, and 10 µl of each solution was dispensed into each 384-cell plate using the MCA 384, to a final concentration of 1 µM and 0.05% of DMSO. The screening was performed in two biological replicates. The same early cell passage was used for all replicate experiments, when the cells had been passaged three times after thawing from liquid nitrogen. A 384-well cell viability assay was optimized for each cell model and implemented using the luminescence detection reagent CellTiter-Glo 2.0 (CTG) Assay kit (Promega, G9243). Units of a luminescent signal generated by a thermo-stable luciferase are proportional to the amount of ATP present in viable cells. Luminescence was recorded using a CLARIOStar (BMG Labtech) (gain = 3600) with bottom reading and cell proliferation was obtained by application of the equation:

$${\mathrm{Inhibition}}\,{\mathrm{Cell}}\,{\mathrm{Proliferation}}\,\left( {{{\mathrm{\% }}}} \right) = 100 - \left( {100 \times \frac{{{\mathrm{compound}}\,{\mathrm{value}}}}{{{\mathrm{median}}\,\left( {{\rm{{DMSO}}}\,{\mathrm{negative}}\,{\mathrm{control}}} \right)}}} \right)$$

### Cell viability assay

HAP1 cell lines, WT or KO MBD4, were treated with several concentrations of gemcitabine (0-1 µM; #S1714, Selleckchem), cytarabine (0-1 µM; #S1648, Selleckchem), doxorubicin (0-1 µM; #S1208, Selleckchem), camptothecin (0–1 µM; #S1288, Selleckchem), paclitaxel (0-1 µM; #S1150, Selleckchem), dacarbazine (0-1 µM; #S1221, Selleckchem), or methytriazine (MTIC; #18863, Cayman Chemical) during 72 h in M199 supplemented with 10% FBS and with or without 2 µM of cytidine precursor (#C4654, Sigma-Merck). MEL202 cell lines, WT or KO MBD4, were treated with several concentrations of gemcitabine, cytarabine, dacarbazine, or MTIC in similar conditions. For the MTT assay, cells were then washed with phosphate-buffered saline (PBS) and incubated with MTT reagent (#CT-02, Merck) for 4 h at 37 °C. The formed formazan crystals were then dissolved in 0.04 N isopropanol-HCl. Absorbance measurement was performed using the FLUOstar OPTIMA plate reader (BMG Labtech) at a wavelength of 570 nm, using 630 nm as reference. For cell count and viability, cells were washed with PBS, and harvested after adding trypsin and after trypan blue staining using the Vi-CELL XR system (Beckman-Coulter). Half-maximal inhibitory concentration (IC_50_) of each cell line for a given drug used was calculated by using GraphPad Prism software (% cell survival = f(log drug concentration)). The experiments were independently performed at least three times.

### Cell death assay

After 24 h of treatment with gemcitabine at IC_50_ cell lines (2 nM in HAP1 cells and 1 nM in MEL202 cells) or cytarabine (0.1 µM in HAP1 cells and 0.2 µM in MEL202 cells), the supernatant was recovered, and adherent cells were harvested with trypsin and counted. The Dead Cell Apoptosis Kit with Annexin V FITC, propidium iodide (#V13242, Thermo Fischer Scientific), and flow cytometer ZE5 Cell Analyzer (BioRad) were used to evaluate the apoptosis rate of cells. The data analysis was done with FlowJo software 10.7.2 version (Supplementary Fig. 4).

### Western blot

HAP1 and MEL202 cells were harvested and washed with PBS. After the incubation in lysis buffer (#9803, Cell signaling technology), cells were sonicated and centrifugated. The supernatant was recovered, and a protein dosage was carried out with a BCA Protein assay kit (#23225, Thermo Fisher). After 4/20% gel migration for 1 h, the gel transfer was carried out with the iBlot 2 Dry Blotting System (Thermo Fisher). The membrane was blocked with 0.1%Tween TBS with 5% milk. Anti-MBD4 primary antibody (1:500) (#ab224809, Abcam) and anti-rabbit HRP-conjugated secondary antibody (1:1000) (#18-8816-31, Rockland) were used. The membrane was scanned with Chemidoc imaging System Biorad (Supplementary Fig. 5).

### Patient-derived xenograft (PDX) treatment

Dacarbazine (Medac) was administered at a dose of 40 mg/kg; Doxorubicin (Teva) was administered at a dose of 1.5 mg/kg; Gemcitabine (Sandoz) was administered at a dose of 160 mg/kg. All these drugs were administered daily intraperitoneally. All in vivo experimental procedures, animal care, and housing were performed in accordance with the recommendations of the European Community (2010/63/UE) for the care and use of laboratory animals and approved by the ethics committee of the Institut Curie CEEA-IC #118 (Authorization APAFiS# 25870-2020060410487032-v1 given by National Authority) in compliance with the international guidelines. For in vivo therapeutic studies, a 15 mm^3^ tumor fragment was grafted into female immunodeficiency SCID mice (Janvier Labs). Relative tumor volumes (RTV) were calculated from the following formula, where Vx is the tumor volume on day x and V1 is the tumor volume on day 1:

$${\mathrm{Relative}}\,{\mathrm{Tumor}}\,{\mathrm{Volume}} = \frac{{Vx}}{{V1}}$$

The statistical significance of differences observed between the individual RTVs corresponding to the treated mice and control groups was calculated by the two-tailed Mann-Whitney test.

### Case report

As described in a previous work^7^, a 76-year-old woman suffering from a metastatic UM was treated first with pembrolizumab. At progression, the patient received standard-of-care dacarbazine (800 mg/m² every 3 weeks) for four months, resulting in progressive disease. After a pause of treatment and because of the positive results observed with the PDX, treatment with gemcitabine was initiated (1000 mg/m² every 2 weeks). In contrast to dacarbazine, gemcitabine treatment resulted in morphological disease control with a metabolic response for 9 months but was stopped because the patient presented symptoms of decompensated heart failure, a rare and already described side effect of gemcitabine. The patient was then aged 81, probably explaining the occurrence of this side effect. Since then, the patient has been followed for 2.5 years with progressive subcutaneous metastases after the interruption of gemcitabine and have been left untreated. This work was conducted in accordance with the declaration of Helsinki and was approved by the Internal Review Board of Institut Curie (Paris, France). The patient provided written informed consent to perform experiments using her fresh and archived tumor samples.

### Statistical analysis

Analyses were carried out with GraphPad software version 5.

### Reporting summary

Further information on research design is available in the Nature Research Reporting Summary linked to this article.

## Supplementary information

Supplementary information

REPORTING SUMMARY

## Data Availability

All relevant data were available upon request to the corresponding author, Dr. Manuel Rodrigues (manuel.rodrigues@curie.fr).
