# Supplementary material for: Cytidine analogs are synthetic lethal with base excision repair default due to MBD4 deficiency
Source: NPJ Precis Oncol. 2022 Nov 2;6:81. doi: 10.1038/s41698-022-00326-z (PMC9630501; doi:10.1038/s41698-022-00326-z)
Supplement: Supplementary file 1 — Supplementary information [file 41698_2022_326_MOESM1_ESM.pdf]

Cytidine analogs are synthetic lethal with base excision repair default due to MBD4 deficiency

Chabot et al.

Supplementary Figures

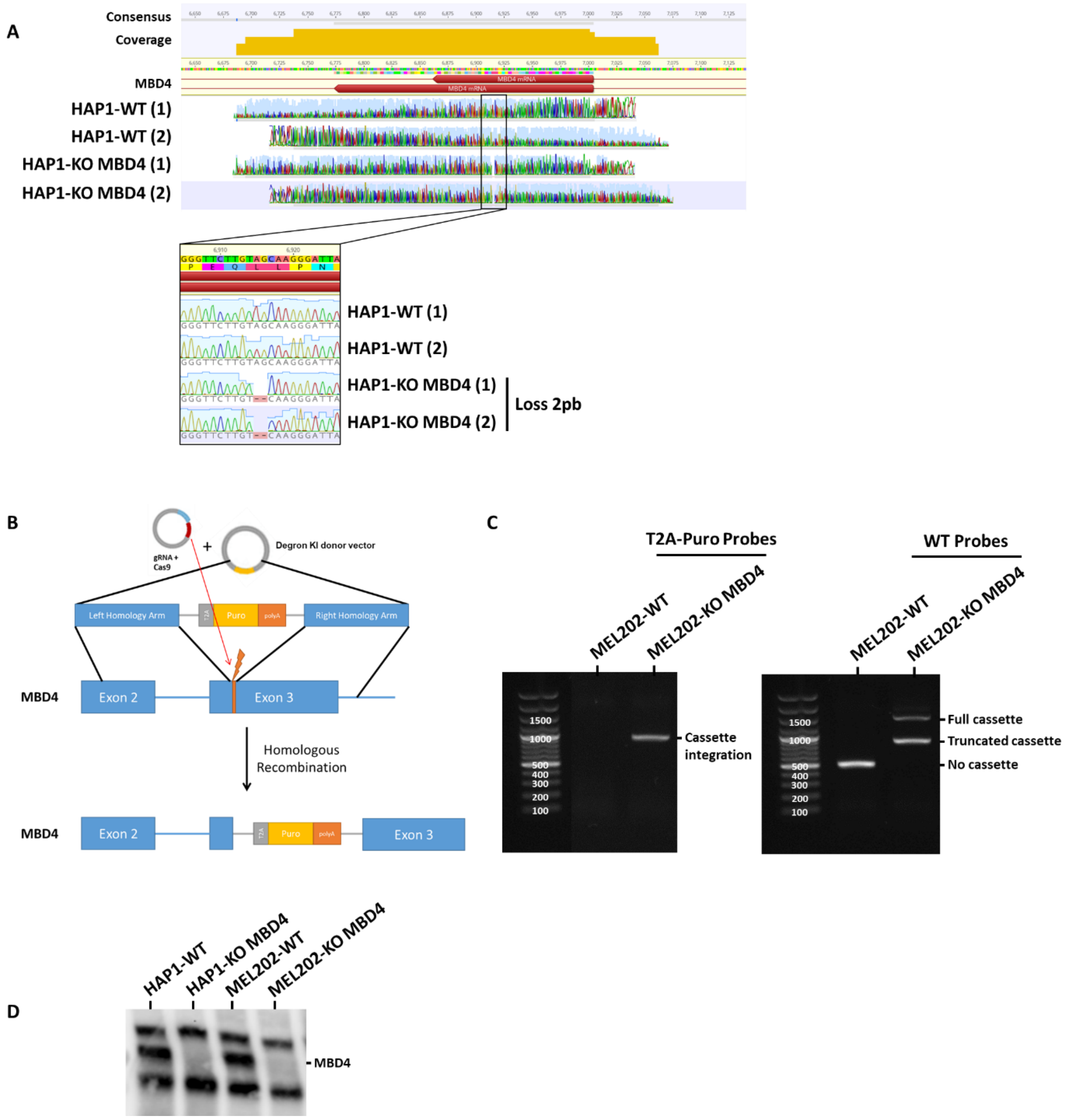

Supplementary Figure 1. Genetic validation of *MBD4* KO cell lines.
(A) Sequencing of the *MBD4* exon 2 gene done in HAP1-WT and KO MBD4 cell lines to confirm the deletion of 2pb in the KO-MBD4 cell line. **(B)** Schematic of the creation of CRISPR KO MBD4 in MEL 202. **(C)** PCR of the puromycin box done in MEL202-WT versus KO MBD4 cell lines to confirm its integration in *MBD4* exon 3 of KO cell lines. **(D)** Western blot in HAP1 and MEL202 cell lines to confirm the loss of MBD4 in KO-MBD4 cell lines. Anti-MBD4 Abcam 224809 (1:500) was used.

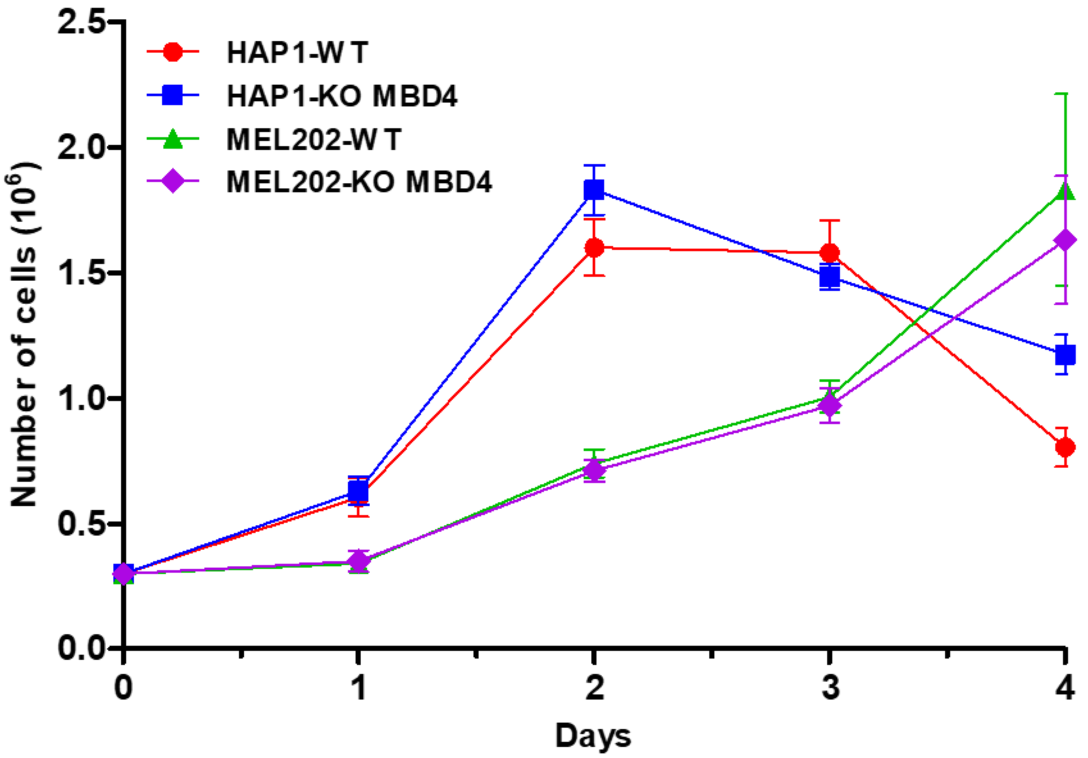

Supplementary Figure 2. Proliferation assays of HAP1 and MEL202 isogenic cell lines. Proliferation assay of HAP1 and MEL202, MBD4-WT and KO, cell lines (n=3, mean ± SD). The difference in number of cells at day 4 between these cell lines is due to the difference in proliferation between these two cell lines. HAP1 are growing much quicker than MEL202, reaching confluence at day 2 (versus day 4).

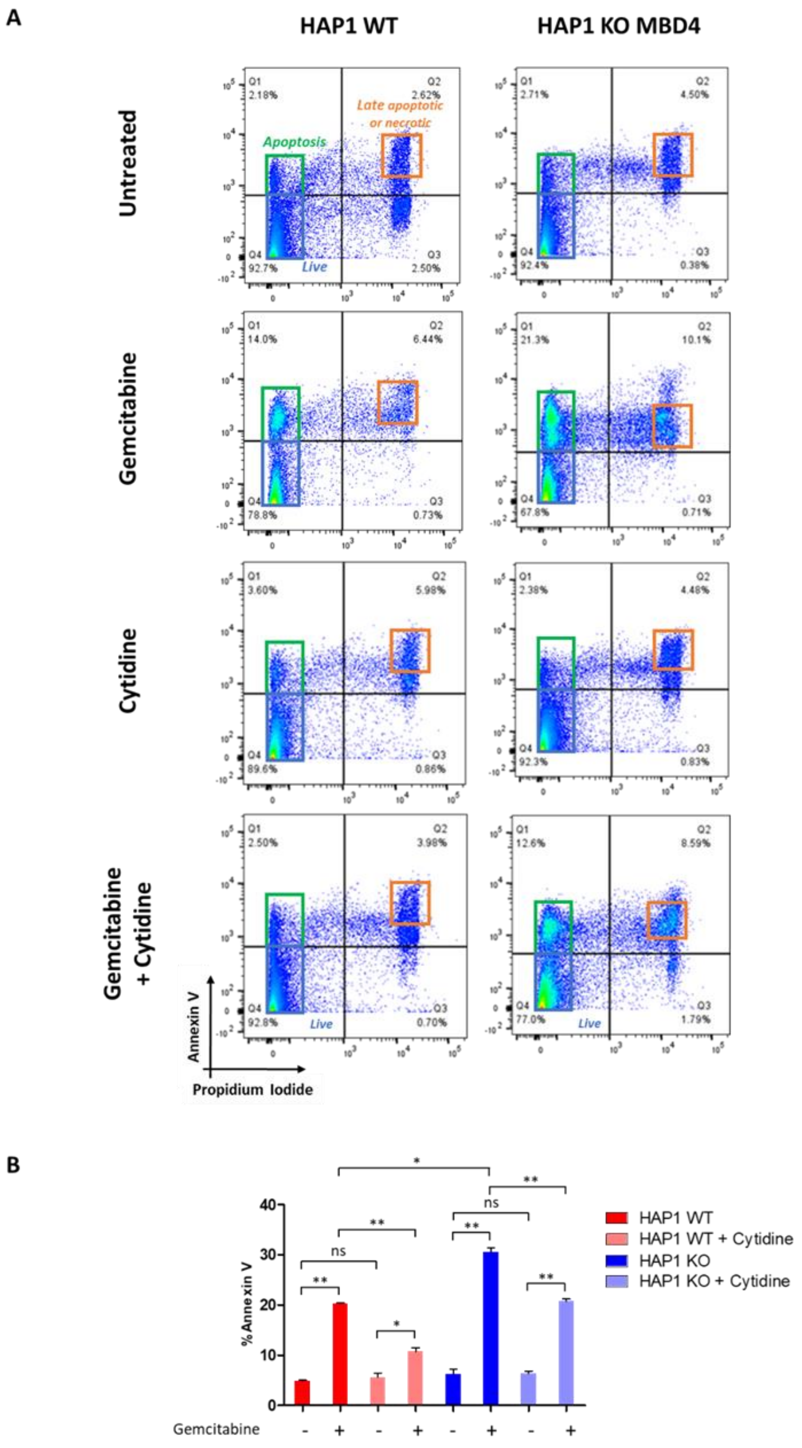

Supplementary Figure 3. Apoptosis assay by flow cytometry of HAP1 cell lines. Supplementation of cytidine.

(A) Scatter plot of HAP1 WT and HAP1 KO MBD4 treated by gemcitabine with or without supplementation with cytidine. **(B)** Percentage of HAP1 MBD4-proficient (red) and deficient (blue) Annexin V positive cells from three independent assays (mean ± SD; n.s : non-significant; *: P < 0.05; **: P<0.01. unpaired two-sided Student's t-test).

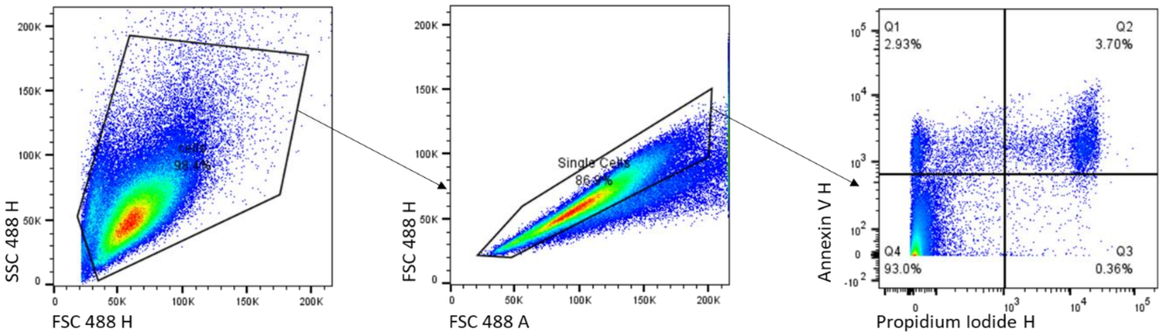

Supplementary Figure 4. Gating strategies used for apoptotic assay.

A

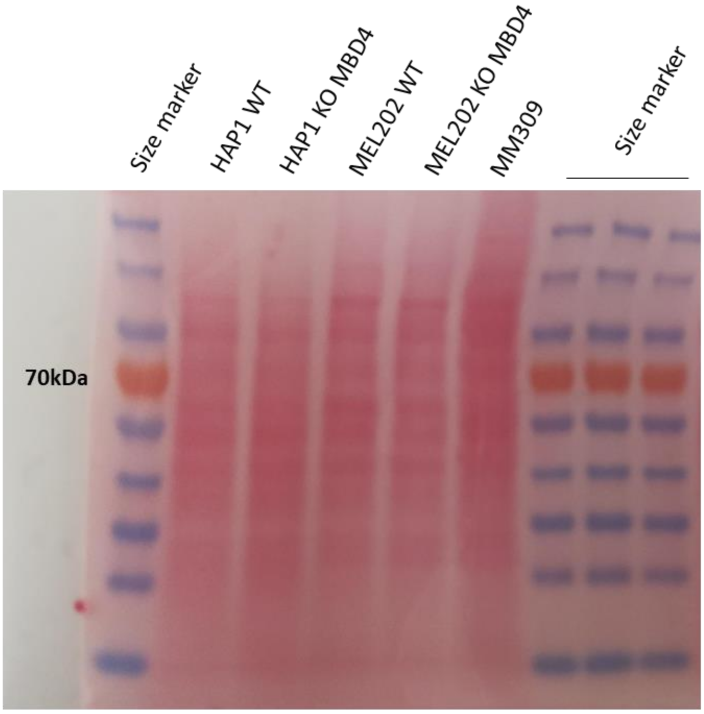

B

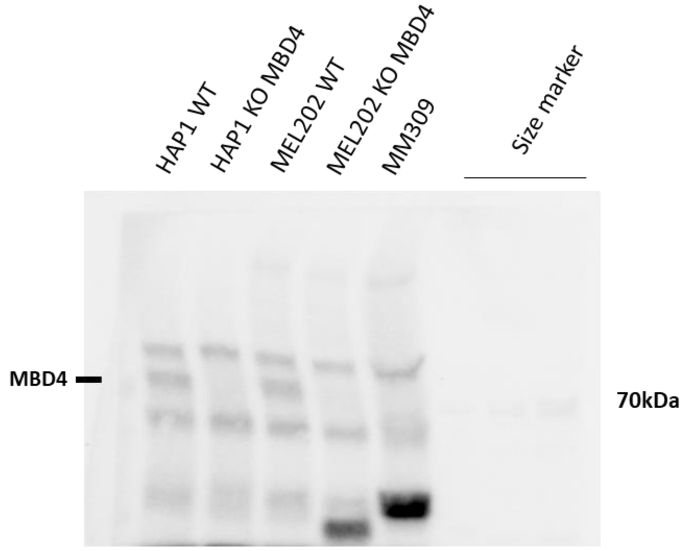

Supplementary Figure 5. Immunoblot MBD4 Raw Data.
(A) HAP1, MEL202 and MM309 protein extracts. Red Ponceau staining as loading control. **(B)** Chemiluminescent Western Blot. Anti-MBD4 primary antibody (1:500) (#ab224809, Abcam), anti-rabbit HRP-conjugated secondary antibody (1:1000) (#18-8816-31, Rockland).

Supplementary Table 1. Top 45 drugs from drug screening inhibiting proliferation in HAP1-KO MBD4 .

Compounds Selected	% Inhibition Proliferation HAP1-WT	% Inhibition Proliferation HAP1-KO MBD4	Drug Family
**daunorubicin hydrochloride**	99,09	99,29	Topoisomerase Inhibitor
**doxorubicin hydrochloride**	99,08	99,26	Topoisomerase Inhibitor
**topotecan**	98,94	99,25	Topoisomerase Inhibitor
**mitoxantrone dihydrochloride**	99,28	99,25	Topoisomerase Inhibitor
**nebularine**	99,28	99,19	Antimetabolite
**(s,+) camptothecine**	99,06	99,17	Topoisomerase Inhibitor
**mitomycin c**	96,81	99,16	Alkylating Agent
**pyrvinium pamoate**	96,04	99,14	Other
**epirubicin hydrochloride**	98,98	99,13	Topoisomerase Inhibitor
**cladribine**	98,76	99,07	Antimetabolite
**gemcitabine**	98,93	99,02	Antimetabolite
**parbendazole**	98,88	98,88	Other
**cytarabine**	96,06	98,88	Antimetabolite
**flubendazol**	98,94	98,83	Other
**podophyllotoxin**	99,11	98,82	Mitotic Spindle Poison
**mebendazole**	98,72	98,81	Other
**lanitoside c**	98,06	98,77	Cardiac Glycoside
**paclitaxel**	99,03	98,76	Mitotic Spindle Poison
**ancitabine hydrochloride**	90,00	98,75	Antimetabolite
**irinotecan hydrochloride trihydrate**	98,31	98,74	Topoisomerase Inhibitor
**nocodazole**	99,10	98,74	Mitotic Spindle Poison
**digoxin**	97,79	98,72	Cardiac Glycoside
**oxibendazol**	98,67	98,68	Other
**docetaxel**	98,50	98,62	Mitotic Spindle Poison
**colchicine**	99,25	98,57	Mitotic Spindle Poison
**albendazole**	98,54	98,37	Other
**fenbendazole**	98,74	97,83	Other
**etoposide**	98,63	97,72	Topoisomerase Inhibitor
**digitoxigenin**	98,40	97,58	Cardiac Glycoside
**gramicidin**	96,67	96,98	Other

Compounds Selected	% Inhibition Proliferation HAP1-WT	% Inhibition Proliferation HAP1-KO MBD4	Drug Family
**salinomycin sodium**	91,02	96,33	Other
**dithiazanine iodide**	91,11	96,27	Other
**trifluridine**	88,78	96,12	Antimetabolite
**monensin sodium salt**	92,41	96,10	Other
**disulfiram**	38,71	95,90	Other
**niclosamide**	92,77	95,63	Other
**raltitrexed**	90,17	93,30	Antimetabolite
**methiazole**	97,67	93,22	Other
**pemetrexed disodium**	91,24	92,87	Antimetabolite
**amethopterin**	90,78	91,26	Antimetabolite
**digoxigenin**	89,99	91,15	Cardiac Glycoside
**thiostrepton**	95,91	89,48	Other
**auranofin**	79,71	87,85	Other
**floxuridine**	90,85	86,86	Antimetabolite
**adapalene**	75,01	83,61	Other
